# Systemic Effects of Ingested Lactobacillus Rhamnosus: Inhibition of Mast Cell Membrane Potassium (IKCa) Current and Degranulation

**DOI:** 10.1371/journal.pone.0041234

**Published:** 2012-07-17

**Authors:** Paul Forsythe, Binxiang Wang, Ibrahim Khambati, Wolfgang A. Kunze

**Affiliations:** 1 Brain-Body Institute, McMaster University, Hamilton, Ontario, Canada; 2 Department of Medicine, McMaster University, Hamilton, Ontario, Canada; 3 Department of Psychiatry, McMaster University, Hamilton, Ontario, Canada; 4 Centre for Simulation-Based Learning, McMaster University, Hamilton, Ontario, Canada; Leiden University Medical Center, Netherlands

## Abstract

Exposure of the intestine to certain strains lactobacillus can have systemic immune effects that include the attenuation of allergic responses. Despite the central role of mast cells in allergic disease little is known about the effect of lactobacilli on the function of these cells. To address this we assessed changes in rat mast cell activation following oral treatment with a strain of Lactobacillus known to attenuate allergic responses in animal models. Sprague Dawley rats were fed with *L.rhamnosus* JB-1 (1×10^9^) or vehicle control for 9 days. Mediator release from peritoneal mast cells (RPMC) was determined in response to a range of stimuli. Passive cutaneous anaphylaxis (PCA) was used to assess mast cell responses in vivo. The Ca^2+^ activated K^+^ channel (KCa3.1) current, identified as critical to mast cell degranulation, was monitored by whole cell patch-clamp. *L.rhamnosus* JB-1 treatment lead to significant inhibition of mast cell mediator release in response to a range of stimuli including IgE mediated activation. Furthermore, the PCA response was significantly reduced in treated rats. Patch-clamp studies revealed that RPMC from treated animals were much less responsive to the KCa3.1 opener, DCEBIO. These studies demonstrate that Ingestion of L.rhamnosus JB-1 leads to mast cell stabilization in rats and identify KCa3.1 as an immunomodulatory target for certain lactobacilli. Thus the systemic effects of certain candidate probiotics may include mast cell stabilization and such actions could contribute to the beneficial effect of these organisms in allergic and other inflammatory disorders.

## Introduction

There is increasing evidence that ingestion of certain non-pathogenic bacteria can modulate local gut mucosal and systemic immune responses to provide potentially therapeutic effects at sites of inflammation and infection [1], [2], [3]. We and several other investigators have identified certain strains of lactobacilli that can reduce lung [1], [4] skin [5] or intestinal [6] allergic inflammation when administered orally. A number of mechanisms have been identified that may contribute to the ability of these bacteria to attenuate allergic inflammation including altered antigen presentation by dendritic cells [7], Th1 polarization [8], [9], or the induction of regulatory T cells [10]. More recently there has been evidence that certain Lactobacilli may influence the effector phase of adaptive inflammation [11].

Mast cells are critical effector cells in a variety of homeostatic and pathological processes [12], [13]. Mast cells are concentrated at interfaces with the external environment, near blood vessels, lymphatic vessels, and nerve fibres. Being positioned at these strategic locations allows the mast cell to act as sentinels and first responders of the immune system, protecting against invading microbes and communicating any change in environment rapidly to the diverse cells involved in physiological and immunological responses [14]. Mast cells are best known for their role in allergic inflammation through the ability of allergen to cross-link allergen-specific IgE bound to the high affinity IgE receptor (FcεR1) expressed on the cell surface [15]. FcεR1 cross-linking triggers a signaling cascade that leads to the influx of extracellular Ca^2+^ and the release of an array of mediators, proteases and cytokines [15]. Despite the central role of mast cells in allergic disease little is known about the effect of anti-inflammatory Lactobacillus species on the function of these cells.

Here we demonstrate that oral treatment with a Lactobacillus strain, *L. rhamnosus* JB-1, previously demonstrated to attenuate the allergic airway response in a mouse model of asthma [1], [16], leads to reduced responsiveness of rat mast cells to an array of degranulating agents. This inhibitory effect on mast cells is associated with decreased membrane potassium current (IK_Ca_) and suggests that action on mast cells may contribute to the anti-allergic effects described for certain commensal bacteria.

## Methods

### Animals

All procedures were conducted in strict accordance with the Guidelines of the Canadian Council on Animal Care All. All procedures were approved by the Animal Research Committee Ethics Board of McMaster University (approval number 08-10-44). Experiments were performed using male Sprague-Dawley rats (Charles River Breeding Laboratories, Saint Constant, QC, Canada) weighing 300–400 g. Rats were housed in the Central Animal Facilities in micro-isolator cages equipped with filter hoods, under controlled temperature (20°C), with a 12∶12 hour light-dark cycle, and free access to food and water.

### Treatment with Bacteria

*L rhamnosus* (JB-1), is the same strain as that employed in several published investigations [1], [17], [18] and was previously referred to as *L reuteri*. This strain was recently confirmed as a *Lactobacillus rhamnosus*, by AFLP fingerprinting and full genomic analysis. It was identified as a strain distinct from L rhamnosus GG [19], and any of the 118 *L. rhamnosus* strains, examined by Vancanneyt et al. [20] and does not belong to any of the 7 clusters identified by the Bacteria Collection Laboratory for Microbiology, University of Ghent, Belgium. *L. salivarius* were a gift from Dr. B. Kiely (Alimentary Health, Cork, Ireland). Both strains were prepared from frozen stocks (–80°C) as described previously [21]. Rats received 1×10^9^ JB-1 or *L.salivarius* in 200 µl of Man-Rogosa-Sharpe liquid medium (MRS broth; Difco Laboratories, Detroit, MI) broth via a gavaging needle daily for 9 days. Control animals were treated daily with 200 µl of MRS broth alone.

### Purification of Rat Peritoneal Mast Cells

Rats were sacrificed by exposure to high concentration of CO_2_, followed by cervical dislocation and exsanguination. Peritoneal mast cells were isolated by injecting 20 ml of ice-cold HEPES-Tyrode's buffer (HTB) into the peritoneal cavity, and the abdomen was massaged for 1 min, opened, and the liquid aspirated into ice-cold polypropylene tubes [22]. Cells were washed by centrifugation (5 min, 150 g, 4°C) and resuspended in 5 ml of HTB. Mast cells were enriched by centrifugation through a discontinuous density gradient of Percoll (>95% purity) [22]. Cell viability was >95% as determined by trypan blue exclusion.

### Measurement of Mast Cell Mediator Release

Purified peritoneal mast cells were suspended at 2.5×10^5^ cells/ml in HTB and stimulated at 37°C to induce β-hexosaminidase release. To test for antigen-induced degranulation, cells were passively sensitized *in vitro* by 4 h incubation with 10 µg/ml mouse monoclonal IgE antibody against the dinitrophenyl haptenic group (anti-DNP) (Sigma, St. Louis, MO), washed twice with the same buffer, then challenged with the antigen, dinitrophenyl-human serum albumin (DNP-HAS) conjugate for 30 min at the stated concentrations. To test for non-IgE mediated activation cells were stimulated with compound 48/80, substance P or the calcium iononphore, A23187 (all from Sigma) at the stated concentrations for 30 min.

β-hexosaminidase was measured in the supernatants and cell pellets, as described previously [23]. Briefly, equal volumes of sample and β-hexosaminidase substrate (1 mM 4-methylumbelliferyl-N-acetyl-β-D-glucosaminide dissolved in dimethyl sulfoxide and 0.2 M sodium citrate) (Sigma) were mixed and incubated for 2 h at 37°C. One hundred microliters of 0.2 M Tris base stopped the incubation. Samples were read using a CytoFluor 2350 fluorescent spectrophotometer at 450 nm (excitation 356 nm). Results are expressed as β-hexosaminidase released as a percentage of total β-hexosaminidase. Measurement of TNF in supernatants of purified peritoneal mast cells was conducted using an ELISA (Abcam, Cambridge, MA) following manufacturers instructions.

### Intracellular Calcium

Changes in intracellular Ca^2+^ following IgE mediated activation of cells was assessed using Fluo-4 NW Calcium Assay Kits (Molecular Probes, Eugene, OR) following manufacturers instructions. Briefly, purified and sensitized RPMC were resuspended in assay buffer to a density of 2.5×10^6^ cells/ml, added to a 96 well plate (50 µl/well) and allowed to settle for 60 min, at 37°C 5% CO_2._ Cells were then incubated with Fluo-4 dye solution for 30 min at 37°C. Fluorescence was measured in all wells (ex 494 nm, em 516 nm) with a Gemini EM Fluorescence Microplate Reader (Molecular Devices, Sunnyvale, CA) to obtain a baseline and then every 20 s following cell stimulation with 100 µM of DNP-HAS for 8 min. Data were analyzed as F/F_0_ (measured fluorescence divided by baseline fluorescence) to adjust for potential differences in baseline florescence.

### Passive Cutaneous Anaphylaxis

Rats were sensitized in the dorsal skin by the intradermal injection of 0.05–0.4 ng/ml anti-DNP IgE. After 24 h, each rat was given 100 µg of antigen (DNP-HSA) with 1% Evans blue via tail vein injection. After 30 min animals were euthanized using CO_2_, the dorsal skin was removed in order to measure the pigment area. The area of leakage of the dye was expressed as the square of the longest and shortest diameters of the blue spot [24]. The skin was cut into pieces and the blue dye was extracted with 5 ml of a mixture of acetone and saline (7∶3) at 37°C overnight. The precipitates were removed following centrifugation at 1700×g for 5 min. Absorbance was then measured at 620 nm.

### Electrophysiology

Mast cells were taken from animals either fed 10^9^ cfu of *L. rhamnosus* JB-1 in broth for 9 days or fed only the same volume of MRS broth. Mast cells were plated onto a 5 ml Petri dish previously coated with poly-L-lysine and containing carbogenated Krebs buffer. After being allowed to settle for 10 min the recording the dish was mounted on an inverted Nikon T-2000 microscope and dish superfused at 1 mL/min with Krebs buffer preheated to 36°C. The Krebs was of the following composition (in mM): NaCl 118.1, KCl 4.8, NaHCO_3_ 25, NaH_2_PO_4_ 1.0, MgSO_4_ 1.2, glucose 11.1, and CaCl_2_ 2.5. Conventional voltage clamp patch clamp recordings were performed as described in Mao et al, a Ca^2+^-current sparing intracellular pipette solution was used to record whole-cell responses to voltage ramp commands. The pipette solution (in mM) was: KMeSO_4_ 110–115, NaCl 9, CaCl_2_ 0.09, MgCl_2_ 1.0, HEPES 10, Na_3_GTP 0.2, and BAPTA.K_4_ 0.2 and 14 mM KOH to bring the pH to 7.3. The IKCa modulating drugs, 5,6- Dichloro-1-ethyl-1,3-dihydro-2H-benzimidazol-2-one (DCEBIO) and 1-[(2-Chlorophenyl)diphenylmethyl]-1H-pyrazole (TRAM-34) were dissolved in DMSO to make stock solutions of 10 mM they were diluted in Krebs to make working concentrations of 1 µM and 5 µM respectively.

We compared the effect of feeding JB-1 in growth medium to feeding only medium (control) on the mast cell I_K,Ca_. Because, for resting mast cells IK_Ca_ channels are essentially closed [25], [26], we used the IK_Ca_ calcium dependent K^+^ channel opener DCEBIO to evoke the current. Outward currents were measured by generating a quasi-steady state I–V relation using a 25 mV/s voltage ramp [27] command ranging from −100 to +80 mV. We used 2 successive ramps [27]: the first was a control with mast cells bathed in Krebs and the second, executed 30 s later, was performed in the presence of DCEBIO. Then, the 2nd trace was subtracted from the 1st, the difference current being that evoked by DCEBIO.

The intermediate conductance calcium activated K^+^ current I_K,Ca_ is not gated by membrane voltage; therefore, the I–V relationship for I_K,Ca_ is governed by the Goldman-Hodgkin-Katz (GHK) flux equation which becomes non-linear when [K^+^] is distributed unequally across the membrane [28]. The DCEBIO current was plotted against command voltage to produce I–V plots. The permeability constant (Ps) for IK_Ca_ diffusion was measured by fitting the I–V plots with the GHK equation:
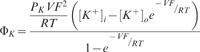
where R, T, and F have their usual meaning, and 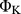 is current density (A/cm^2^) and V is the membrane potential (V) [28], [29]. We expressed P_K_ in cm^3^/s rather than cm/s because we recorded the IK_Ca_ current (I_K,Ca_) (A) rather than 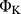.

### Statistics

Experimental results are expressed as means ± standard deviations. Data were analyzed using the Student t test or One way analysis of variance (ANOVA) with a Tukey post-hoc test. For measurements of electrophysiological data, current subtractions and I-V relation plotting were made using Clampfit 10 (Molecular Devices). Curve fits and descriptive or comparative statistics were calculated using GraphPad Prism 5.0 (GraphPad Software, San Diego, CA, USA). The statistically discernable difference for comparative tests was set at P = 0.05.

## Results

### Oral Administration of L. rhamnosus JB-1 Inhibits Degranulation of Rat Peritoneal Mast Cells

**Figure 1:**
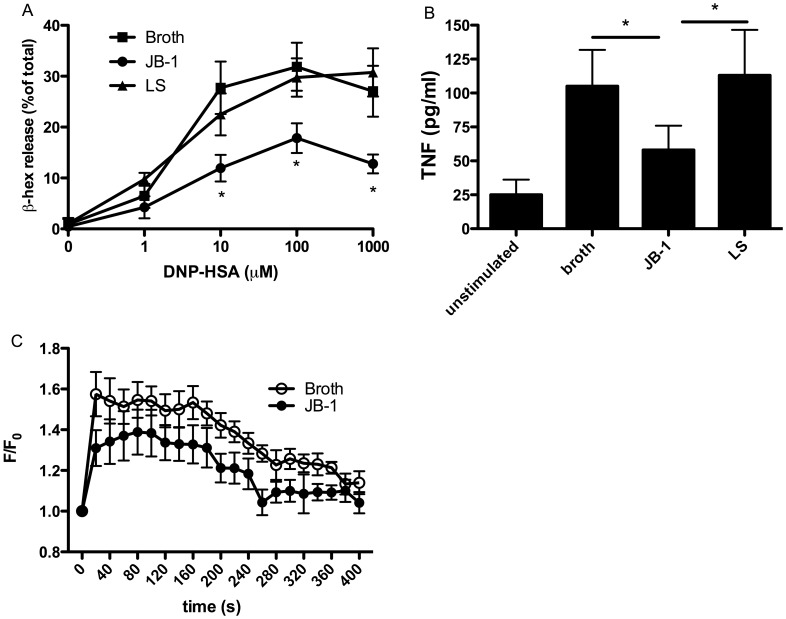
IgE mediated β-hexosaminidase (A) and TNF (B) release from rat peritoneal mast cells isolated following 9 days oral treatment with MRS (Broth), *L. salivarius* (LS) or *L.rhamnosus* JB-1 (JB-1). β-hexosaminidase is expressed as percentage of total β-hexosaminidase in the cells. The intracellular Ca^2+^ response to stimulation with 100 µM DNP-HSA (C) is expressed as fluorescence following stimulation divided by baseline fluorescence (F/F_0_ ). Data is presented as mean ± SD, n = 6 animals in each treatment group from 3 independent experiments, * = p<0.05.

**Figure 2:**
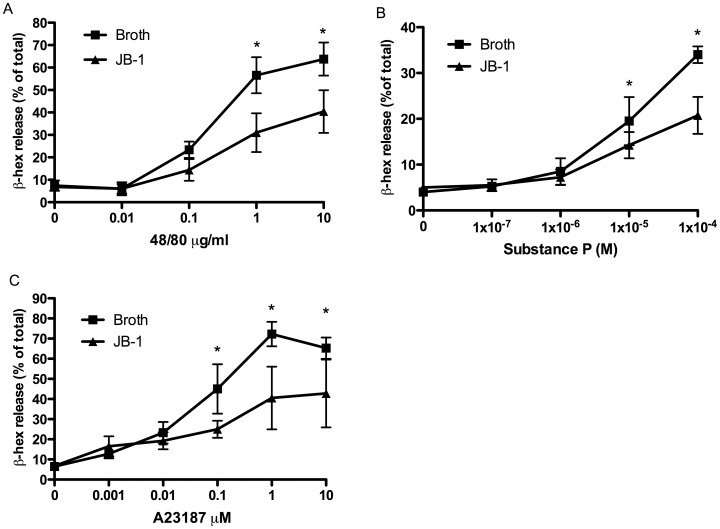
β-hexosaminidase release from rat peritoneal mast cells isolated following 9 days oral treatment with MRS (Broth) or *L.rhamnosus* JB-1 (JB-1) in response to 48/80 (A), Substance P (B) and A23187 (C). β-hexosaminidase is expressed as percentage of total β-hexosaminidase in the cells. Data is presented as mean ± SD, n = 6 in each treatment group, 3 independent experiments * = p,0.05.

Feeding of JB-1 lead to significant inhibition of IgE mediated degranulation, of isolated peritoneal mast cells as assessed by release of β-hexosaminidase (41.8±3.8% decrease in maximal release, n = 6 *P*<0.01). Feeding with JB-1 also inhibited TNF release from the peritoneal mast cells (Fig. 1A). This inhibitory action was species and strain specific as feeding of the same number of *Lactobacillus salivarius* did not alter the responsiveness of RPMC to IgE mediated activation (Fig. 1B). Furthermore, the inhibition of degranulation was associated with a decreased intracellular calcium response to IgE mediated stimulation (Fig. 1C) with a maximal F/F_0_ of 1.58±0.10 and 1.39±0.11 for RPMC from broth and JB-1 treated mice respectively (n = 6, p = 0.014). To determine if the inhibitory effect of feeding JB-1 was specific to IgE mediated release we also assessed the response of isolated RPMC to a range of other stimuli. As with FcεR1 receptor activation, mast cells isolated from JB-1 treated animals demonstrated a reduced response to the neuropeptide substance P (49.0±12.9% decrease in maximal release, *n* = 6 *P*<0.01) and the non-immunological stimuli 48/80 (34.4±9.7% decrease in maximal release, *n* = 6 *P*<0.01) and the calcium ionophore A23187 (43.6±18.5% decrease in maximal release, *n* = 6 *P*<0.01) (Fig. 2).

### Co-culture of Mast Cells with L. rhamnosus JB-1 does not Inhibit Degranulation

**Figure 3:**
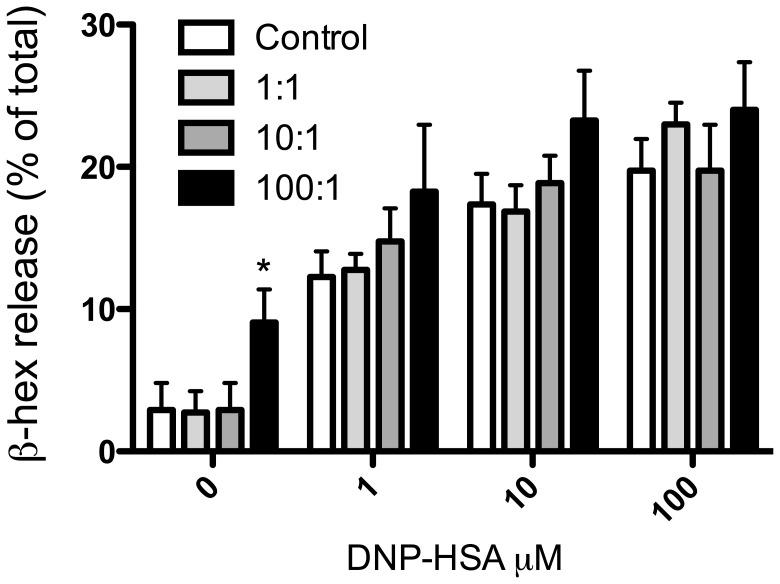
The effect of *in vitro* co-culture of *L.rhamnosus* JB-1 with rat peritoneal mast cells isolated from untreated rats. Passively sensitized RPMC were co-cultured with bacteria at a ratio of 1∶1, 10∶1 and 100∶1 (bacteria:mast cell) for 8 hours prior to activation. β-hexosaminidase is expressed as percentage of total β-hexosaminidase in the cells. Data is presented as mean ± SD, n = 5 independent experiments * = p<0.05.

To determine whether *L. rhamnousus* JB-1 could directly modulate mast cell activity we conducted *in vitro* co-culture studies with RPMC isolated from untreated rats. Passively sensitized RPMC were co-cultured *in vitro* with JB-1 at a ratio of 1∶1, 10∶1 and 100∶1 bacteria: RPMC for 8 hours prior to activation. The *in vitro* exposure of RPMC to JB-1 did not attenuate subsequent degranulation in response to antigen exposure (Figure 3). Indeed, at a ratio of 100 JB-1 per mast cell there was a small but statistically significant increase in β-hexosaminidase release from the mast cell in the absence of antigen.

### Oral Administration of L. rhamnosus JB-1 Attenuates Passive Cutaneous Anaphylaxis

**Figure 4:**
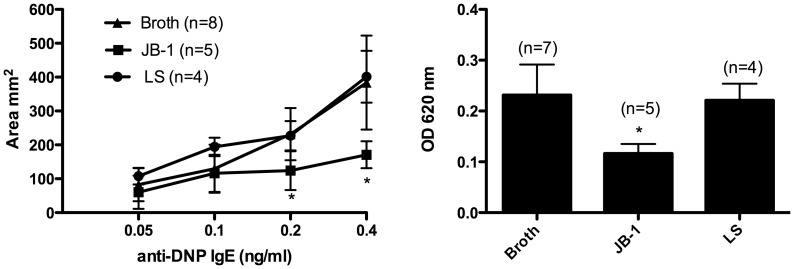
The passive cutaneous reaction induced by DNP-HSA following sensitization with anti-DNP IgE in rats treated orally with MRS broth (broth), *L.rhamnosus* (JB-1) or *L. salivarius* (LS) as assessed by the reaction area (A) and the amount of dye extracted from the back skin (B). Data is presented as mean ± SD, 3 independent experiments were performed with number of animals in each treatment group as indicated, * = p<0.05.

Given that the mast cell stabilizing effect of JB-1 treatment did not appear to require direct interaction between bacteria and cell, we wanted to determine whether mast cells beyond the peritoneal cavity were stabilized and if there was an *in vivo* consequence of such stabilization. The passive cutaneous anaphylaxis (PCA) reaction is a well-documented model for mast cell-dependent immediate hypersensitivity and can be used to assess anti-allergic activities of compounds [30], [31]. When orally administered for 9 days, JB-1 produced marked inhibitory effects on the PCA reaction induced by DNP-HSA following sensitization with anti-DNP IgE as assessed by the reaction area and the amount of dye extracted (Figure 4 A&B). In keeping with our findings for IgE mediated degranulation of RPMC, 9 days treatment with 1×10^9^ cfu of *L. salivarius* did not significantly modulate the PCA response.

### KCa3.1 Channel Opening on Rat Peritoneal Mast Cells is Inhibited

**Figure 5:**
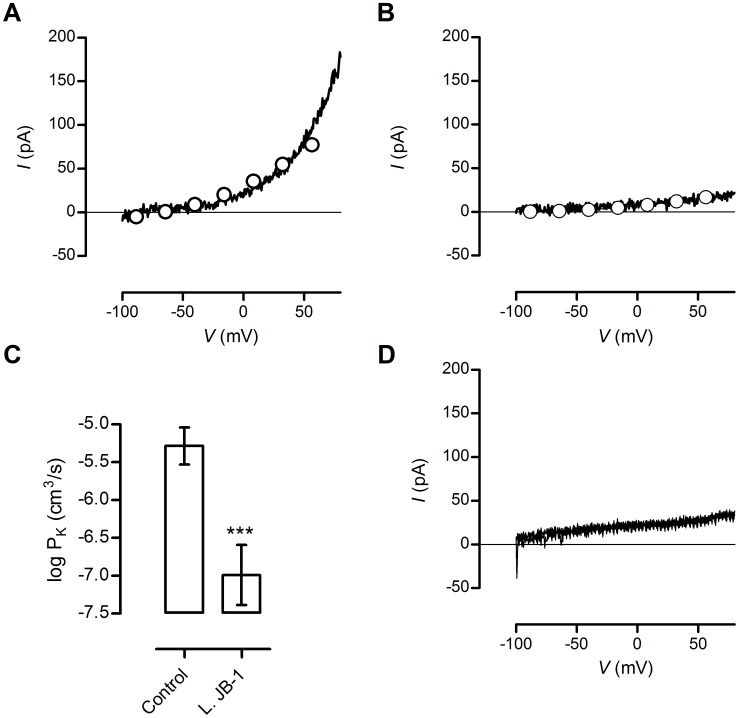
Current – voltage relations for DCEBIO evoked currents in mast cells from *L.rhamnosus* JB-1 fed or control animals. A) I–V plots of DCEBIO current recorded from mast cell of control animals. The current was fitted with the GHK equation (O) and PK for this cell was determined to be 5.1×10^−6^ cm3/s. B) DCEBIO current recorded from mast cell of *L.rhamnosus* JB-1 fed animal was substantially smaller than that from control animals. For this representative cell PK  = 1.3×10^−7^ cm^3^/s. C) Summary data for DCEBIO current experiments given as mean ± SEM. Feeding *L.rhamnosus* JB-1 instead of culture medium substantially blocked the DCEBIO current (P = 0.0001). D) Two superimposed traces of I-V plots, the first was performed only Krebs buffer as the perfusate and the second was made when 1 µM DCEBIO was added in the presence of 5 µM TRAM-34. DCEBIO had no effect in the presence of the IKCa channel blocker TRAM-34, cells were obtained from at least 4 animals in each treatment group, *** = p<0.001.

Given our previous studies indicating that JB-1 may modulate the activity of the intermediate conductance calcium dependent potassium current (IK_Ca_) channel in enteric sensory neurons, and the evidence that opening of this ion channel plays an important role in mediating mast cell degranulation, we assessed the electrophysiological response of mast cell to the presence of a specific IK_Ca_ channel opener DCEBIO. In mast cells from vehicle fed animals, DCEBIO evoked a strong positive current whose outward rectification was evident from the I–V plots (Fig. 5A) which were well fitted (O) by the GHK equation for a K^+^ current giving P_K_ of 5.7×10^−6^±2.0×10^−7^ cm^3^/s (n = 10). We demonstrated the specificity of the DCEBIO evoked current by showing that addition of an IK_Ca_ channel inhibitor, TRAM-34 (5 µM) to the superfusate 20 min before DCEBIO was added prevented the induction of an outward current (Fig. 5D). Since TRAM-34 is highly selective for the IK_Ca_ current, it is likely that DCEBIO selectively opened IK_Ca_ channels. Mast cells from JB-1 fed animals exhibited a reduced DCBIO induced current (Fig. 5B), so that P_K_ was decreased to 1.8×10^−7^±2.9×10^−7^ cm3/s (*n* = 12) (Fig. 5C) (P = 0.0001, Mann-Whitney test, 2-tailed). Thus, feeding JB-1 reduced the IK_Ca_ channel opening in mast cells similar to the proposed mode of action of JB-1 on enteric sensory neurons [17].

## Discussion

We have demonstrated that oral treatment with *L.rhamnosus* JB-1 leads to the stabilization of mast cells in the peritoneal cavity and skin, thus providing further evidence of the capacity of this bacterium for systemic immunomodulation [1], [16]. Furthermore, these studies identify inhibition of IK_Ca_ as a common component of the modulatory action of JB-1 on enteric sensory neurons [32] and mast cells.

The ability of certain strains of bacteria, generally Lactobacillus or Bifidobacterium to attenuate allergic inflammation has been established in animal models of asthma, atopic dermatitis and food allergy [1], [4], [6]. These model systems in conjunction with *in vitro* studies have suggested a number of mechanisms that seem to contribute to microbial-induced attenuation of allergic inflammation. Such mechanisms include altered antigen presentation by dendritic cells and subsequent decrease in IgE responses [7], a skewing of T cell polarization towards Th1 responses [8], [9], and the induction of regulatory T cells [10], [16], [33]. Our current findings suggest that inhibition of mast cell activation may also contribute to anti-allergic effects following oral treatment with certain bacteria.

There have been recent reports that other candidate probiotic bacteria can attenuate mast cell degranulation [34]–[36] and one report links this effect to a decreased adoptive anaphylaxis response in mice [35]. However, in contrast to our current data, these previous studies administered the bacteria *i.p*. and indicated that direct interaction between mast cell and bacteria mediated the inhibitory effect. In contrast, JB-1 effectively stabilized peritoneal mast cells following feeding, while direct *in vitro* co-culture with JB-1 did not influence mast cell response to stimuli. This suggests an indirect mechanism of action involving additional cell types. It has also been reported that direct exposure of human peripheral blood mast cells to a *Lactobacillus rhamnosus* strain lead to a downregulation of FcεR1 expression on the cell surface. We did not assess FcεR1 expression on mast cells from JB-1 fed animals. However, as treatment with JB-1 also inhibits degranulation in response to non-IgE mediated activation it is unlikely that a changes in expression of this receptor account for the inhibition of degranulation observed.

Significantly, we identified that oral treatment with JB-1 leads to an inhibition of an intermediate calcium-dependent potassium channel (IK_Ca_) current in peritoneal mast cells. We have previously demonstrated that JB-1 selectively increases the excitability of myenteric AH/Dogiel type II neurons as demonstrated by a decreased threshold for activation as well as an increased number of action potentials generated upon depolarization [17]. This increase in excitability was attributed to a decreased slow afterhyperpolarization caused by a reduction in IK_Ca_ current, an effect mimicked by the KCa3.1 blocker TRAM-34 [17]. While blocking IK_Ca_ increases excitability of myenteric AH neurons it has previously been demonstrated to decrease mast cell response to stimuli [26], [37].

The activity of K^+^ channels maintains the cell membrane potential that acts as the electrical driving force for Ca^2+^ entry, required for mast cell degranulation. Activation of K^+^ channels, with subsequent hyperpolarization of the cell membrane, enhances mast cell degranulation, because cell membrane Ca^2+^ influx is greater at negative membrane potentials [38]. Consequently, inhibiting K^+^ channels or decreasing the drive for K^+^ exit inhibits mast cell degranulation [26], [39], [40]. Of the K+ channels expressed in mast cells, Ca^2+^-activated K^+^ channels, specifically IK_Ca_ (KCa3.1) have been implicated in the regulation of exocytosis [26], [37]. Indeed it has been demonstrated that KCa3.1 opening is not required for, but potentiates, mast cell secretion [26], [37]. The IK_Ca_ opener 1-EBIO enhances IgE-dependent Ca^2+^ influx and degranulation in response to a submaximal stimulus [26] while mice from KCa3.1 deficient (KCa3.1−/−) demonstrate attenuated degranulation in response to Fcer1 mediated activation [37]. In keeping with this our studies indicate that JB-1 mediated inhibition the IKCa3.1 was associated with a decreased intracellular calcium response activation. IgE-dependent degranulation is not the only KCa3.1-dependent process in mast cells. Stimulation of mast cells via endothelin receptors was also impaired in KCa3.1−/− BMMCs [37] and activation of the channel is critical to the migration of human lung mast cells [41]–[43]. It is therefore likely that the observed inhibition of IK_Ca_ current in mast cells obtained from *JB-1* fed animals is responsible at least in part for the decreased degranulation of these cells in response to a range of stimuli. In this regard, it is interesting to note that the degree of attenuation in response to IgE mediated activation is similar to that observed in mast cells from KCa3.1−/− mice [37].

The mechanism through which JB-1 feeding leads to inhibition of IK_Ca_ current is currently unknown. The activation of a range of receptors on the mast cell surface including, β_2_-adrenoceptors, A_2A_ adenosine receptors and EP2 prostaglandin receptors can lead to inhibition of the IKCa current [25], [42], [43]. A commonality between these receptors is they are G_s_-coupled and therefore it is possible that other G_s_-coupled receptors may also inhibit KCa3.1 opening. Thus a range of immune or neuronal derived mediators could be responsible for JB-1 induced inhibition of mast cells.

Overall, these results suggest that inhibition of mast cell responses may be a component of the systemic immunomodulatory effects of commensal bacteria and a contributing factor to the ability of certain candidate probiotic organisms to attenuate allergic inflammation. Future studies will focus on potential mediators and corresponding receptors responsible for mast cell stabilization. The KCa3.1 channel current has been identified as critical to the function of many immune cells [44]–[47] and has been proposed as a therapeutic target in a range of immune disorders including allergy [48], [49]. Thus it will be interesting to determine if the channel’s function is altered in other cell types and whether inhibition of KCa3.1 may contribute to a number of the diverse physiological effects described for certain commensal organisms.
